# Transparent RFID tag wall enabled by artificial intelligence for assisted living

**DOI:** 10.1038/s41598-024-64411-y

**Published:** 2024-09-16

**Authors:** Muhammad Zakir Khan, Muhammad Usman, Ahsen Tahir, Muhammad Farooq, Adnan Qayyum, Jawad Ahmad, Hasan Abbas, Muhammad Imran, Qammer H. Abbasi

**Affiliations:** 1 James Watt School of Engineering, University of Glasgow, Glasgow, G12 8QQ UK; 2 Artificial Intelligence Research Centre, Ajman University, Ajman, UAE; 3 School of Computing, Engineering and Built Environment, Glasgow Caledonian University, Glasgow, G4 0BA UK; 4 School of Computing, Edinburgh Napier University, Edinburgh, UK; 5 Electrical engineering department, Information Technology University, Lahore, Pakistan

**Keywords:** Health care, Engineering, Mathematics and computing

## Abstract

Current approaches to activity-assisted living (AAL) are complex, expensive, and intrusive, which reduces their practicality and end user acceptance. However, emerging technologies such as artificial intelligence and wireless communications offer new opportunities to enhance AAL systems. These improvements could potentially lower healthcare costs and reduce hospitalisations by enabling more effective identification, monitoring, and localisation of hazardous activities, ensuring rapid response to emergencies. In response to these challenges, this paper introduces the ***T****ransparent* ***R****FID* ***T****ag Wall (TRT-Wall)*, a novel system taht utilises a passive ultra-high frequency (UHF) radio-frequency identification (RFID) tag array combined with deep learning for contactless human activity monitoring. The *TRT-Wall* is tested on five distinct activities: sitting, standing, walking (in both directions), and no-activity. Experimental results demonstrate that the *TRT-Wall* distinguishes these activities with an impressive average accuracy of $95.6\%$ under four distinct distances (2, 2.5, 3.5 and 4.5 m) by capturing the RSSI and phase information. This suggests that our proposed contactless AAL system possesses significant potential to enhance elderly patient-assisted living.

## Introduction

Human activity recognition (HAR) plays a significant role in facilitating remote health monitoring and activity-assisted living (AAL) for elderly individuals wishing to maintain independence at home. With an aging population, the need for such assistance is growing. United Nations estimates^1^ predict a decrease in the ratio of individuals aged 15 to 64 to those over 65 from 7:1 in 2020 to 4:1 by 2050, leading to a global elderly population of approximately 2 billion by 2050^2^. This demographic trend suggests a potential workforce shortage in elderly care, emphasising the importance of AAL research^3^. AAL integrates various technologies to support carers by addressing challenges such as limited mobility, chronic disease monitoring, reducing social isolation, and managing medication^4^. The medical data collected from these technologies is essential for the growing demand for technology-driven healthcare solutions, particularly for disabled patients in indoor environments^5^. The motivation for this study is to develop affordable and innovative monitoring technologies to improve the quality of life and empower elderly individuals to live independently. Despite progress in HAR and AAL systems, current solutions often face challenges such as high costs, privacy issues, and the inconvenience of wearable sensors. The proposed *TRT-Wall* system aims to address these challenges using commercially available off-the-shelf (COTS) ultra-high frequency (UHF) RFID technology. This study focuses on providing a cost-effective, contactless, and easy-to-install solution that bridges the gap between advanced HAR technologies and practical applications for elderly care. This approach will enhance our ability to monitor and support the elderly, promote their independence and safety, and contribute significantly to the growing field of AAL.

In recent years, HAR has been utilised by camera systems or on-body sensors such as infrared, accelerometers/gyroscopes, and frequency-modulated continuous wave (FMCW) radar^6,7^. However, these systems face challenges^8,9^. For example, camera-based monitoring can encounter issues like occlusion, restricted perspective, low lighting and frame resolution, and high computational demands for video processing. Privacy concerns are also significant, although studies indicate a willingness among the elderly to trade some privacy for increased autonomy^8^. Wearable sensors can be burdensome during activities like sleep or physical exercise, with a risk of users forgetting or losing interest in wearing them^10^. As an alternative, contactless (tag-free) sensors using technologies like Wi-Fi, Radar, and RFID offer benefits such as enhanced privacy and better performance in complex indoor environments with various obstacles and moving objects, creating multiple signal paths^11^. While radar-based solutions with large antenna systems and wide bandwidths have been successful in accurate, real-time activity monitoring, they are often expensive, power-hungry, and not widely accessible^12^. A more economical option is to use UHF RFID readers in combination with battery-less and compact RFID tags.

RFID technology is a practical and cost-effective solution for remotely monitoring elderly healthcare^13^. Its benefits include low cost, compact size, scalability, shareability, and battery-free operation^14^. Recent advances have produced inexpensive, highly sensitive passive tags with read ranges exceeding 10 meters, supporting their use as an economically viable solution for pervasive healthcare^5^. The emergence of ‘tag-free’ sensing, which employs contactless technology instead of attaching tags directly to the human body^15^, presents a potential solution for AAL challenges^16^. This approach is less cumbersome and invasive for recognising fundamental activities like standing, sitting, running, and walking, which are essential for well-being. These systems detect target objects or events by analysing signal characteristic changes, such as RSSI and phase shift. Commonly, coarse-grained measurements of RSSI and phase value are used for sensing^17^, but accurate results for indoor AAL and positioning require consideration of factors such as obstacles causing non-line-of-sight (NLOS) conditions, signal weakening due to rapid fading, and multipath effects from indoor construction materials, along with climate changes impacting signal propagation speed. These factors collectively affect the accuracy and reliability of AAL and positioning systems in indoor environments^18^.

This study introduces a monitoring system utilising COTS UHF RFID technology, operating within the frequency range of 860 to 950 MHz. The primary objective is to develop intelligent walls equipped with RFID tag arrays on each side to distinguish five distinct human activities: sitting, standing, walking in two directions, and no-activity. Our proposed *TRT-Wall* system hypothesises that the presence and movement of the human body within the radio field will result in recognisable RFID signal patterns due to attenuation, diffraction, reflection, and multipath effects. Notably, the *TRT-Wall* approach enables monitoring the daily activities of elderly patients using pseudo-localisation, reusing low-cost printed RFIDs and existing RFID readers for indoor activity recognition. Additionally, it ensures simple deployment using COTS RFID readers, requiring only a single reader with a single antenna.

Specific contributions of this paper are:

1. We propose *TRT-Wall*, which uses contactless UHF RFID tags for sequential and simultaneous activity detection. Specifically, we collected a dataset for four different activities: sitting, standing, and walking (forward and backward).
2. The propose *TRT-Wall* leverages RSSI and phase data fluctuations for activity localisation.
3. We perform an extensive evaluation of the collected dataset to determine the walking direction (i.e., forward or backward).
4. We calculate the speed of the moving object to establish a relationship between detection and activity location.

The paper is structured into several sections. Section “Related work” discusses related studies pertinent to the problem. Section “Evaluation and results” presents data evaluation strategies and the obtained results. Section “Discussion” analyses the results in detail. Section “Data and methods” explains data sources and research methodologies. Section “Limitations and future directions” discusses the study’s limitations and suggests directions for future research. Finally, Section “Conclusion” provides the paper’s concluding remarks.

## Related work

In HAR, researchers have increasingly focused on utilising UHF passive RFID tags to improve the quality of life for the elderly. These applications range from location and mobility monitoring to medication management and fall prevention. Both ‘tag-free’ and ‘tag-based’ technologies have been explored for tracking and analysing daily activities. For example, Raad et al.^19^ introduced a ‘tag-based’ prototype using passive RFID wearable anklets or bracelets to detect wandering elderly individuals within their homes. Shuaieb et al.^20^ proposed a low-cost indoor location system based on RFID tags to enhance automated alarm systems in nursing homes and to trigger emergency services for stationary targets. Feng et al.^21^ developed a posture-recognition system named ‘SitR’, which uses RF signals and three lightweight RFID tags on the user’s back to recognise seven sitting postures. Systems like ‘TagCare’, a fall detection system for the elderly, utilise RSSI and Doppler frequency readings^22^. Another suggested approach for fall detection employs passive RFID sensor tags in indoor footwear, monitoring RSSI and pressure changes. Toda et al.^23^ provided a comprehensive mechanism for fall detection by analysing routine activities through shoe sole pressure data and RSSI fluctuations. Ruan et al.^24^ introduced the ‘TagFall’ system, which uses abrupt changes in RSSI values to distinguish falls from daily activities. These methods demonstrate the potential of passive ‘tag-based’ solutions for recognising various activities and postures.

‘Tag-free’ sensing offers several advantages for AAL, including low cost, non-intrusive nature, and structural simplicity. Sigg et al.^25^ introduced a ‘tag-free’ radio-based activity detection method using ambient FM radio signals. He et al.^26^ proposed a technique to enhance the signal-to-noise ratio of RFID tags for detecting activities such as arm swings and knee bends without physical contact. Zou et al.^27^ developed the ‘GRfid’ system for gesture recognition, employing multi-tag phase measurement and normalising dynamic time warping (DTW) distance. Dian et al.^28^ introduced the ‘RFree-GR’ system, capable of recognising fine-grained gestures, evidenced by its evaluation of 16 American Sign Language words. Zhao et al.^29^ presented the ‘RF-Motion’ system, which uses RFID technology to identify six types of human motion with an $87\%$ accuracy rate, utilising DTW, synthetic aperture algorithms, and data slicing. RFID tag arrays have been used to predict motion and pose-based activities, with challenges such as temporary occlusions or physical obstructions in wall-mounted systems^30,31^. The ‘RF-HMS’ system employs an RFID tag array to monitor tag-free human motion through walls, identifying the presence or absence of moving individuals with an average accuracy of 90%^32^. The ‘IDSense’ system integrates passive RFID tags into everyday objects to detect human-object interactions, including motion and touch, primarily in proximity to walls^33^.

Previous ‘tag-free’ approaches primarily rely on tag or tag arrays as reference points to detect object motion or direction, or they require proximity to the tags for activity recognition, thus limiting their scope. In contrast, the *TRT-Wall* approach facilitates AAL monitoring of elderly patients through pseudo-localisation, utilising a cost-effective printed RFID array in conjunction with a single COTS reader and antenna. Table 1 outlines the contrasting features among different existing systems.

**Table 1:** Comparison of existing systems in human activity recognition.

System	Activity recognised	Tag-based or tag-free	Algorithm utilised
Raad et al.^19^	Wandering detection	Tag-based (anklets/bracelets)	RSSI Fingerprinting
Shuaieb et al.^20^	Indoor localisation	Tag-based (head to ankle)	Euclidean RSSI distance
Feng et al.^21^	Posture recognition	Tag-based (on back)	Machine learning
Zhu et al.^22^	Fall detection	Tag-based (on neck)	RSSI, Doppler frequency
Toda et al.^23^	Fall detection	Tag-based (in shoe)	Machine learning (RSSI pressure)
He et al.^26^	Activity detection	Tag-free (single antenna and tag)	Enhanced signal-to-noise
Dian et al.^28^	Gesture recognition	Tag-free (distance between tags 70–90 cm)	Machine learning
Wang et al.^34^	In-Car activity	tag-free (four antennas and six tags)	Adversarial networks (RSSI)
Zhao et al.^29^	Motion identification	Tag-free (four antennas and six tags)	DTW, SAR (RSSI and phase)
Oguntal et al.^31^	Motion and pose prediction	Tag-free (two antennas and 228 tags)	Machine learning
TRT-Wall	Motion and activities	Tag-free (1 antennas and 15 tags)	Machine learning

## Evaluation and results

This section presents the results of four distinct experimental scenarios, each involving different subjects performing various activities, as depicted in Fig. 4. The evaluation includes an assessment of both the overall performance and the impact of reader-subject and subject-tag distances, as well as the type of activity on the system’s accuracy. Tables 2 and 3 provide a comprehensive evaluation used to assess the system’s robustness.

**Table 2:** Deep learning classification accuracy in multi-distance and multi-subject.

Subjects	4.5 m			3.5 m			2.5 m			2 m		
	RSSI (%)	Phase (%)	FeatureSet (%)	RSSI (%)	Phase (%)	FeatureSet (%)	RSSI (%)	Phase (%)	FeatureSet (%)	RSSI (%)	Phase (%)	FeatureSet (%)
LSTM accuracy												
1	**81.2**	68.3	75.2	**87.4**	81.2	62.5	60.6	62.7	**74.1**	53.5	62.9	**69.5**
2	**91.6**	75.2	88.6	**91.8**	87.7	85.7	72.4	68.5	**79.8**	66.8	66.3	**73.4**
3	**93.7**	92.1	90.3	**95.6**	94.3	91.6	76.4	73.5	**82.4**	70.2	70.3	**76.4**
CNN accuracy												
1	**78.7**	75.3	78.1	68.2	**86.6**	81.4	63.4	**66.5**	61.3	60.3	66.5	**70.4**
2	**82.3**	81.3	79.5	**89.5**	88.4	87.6	**69.3**	67.5	64.3	64.8	70.4	70.4
3	89.3	**93.2**	85.7	**93.5**	92.6	87.5	76.5	76.6	**84.4**	73.7	74.5	**76.3**
LSTM+CNN accuracy												
1	**79.6**	75.7	78.4	**86.6**	68.7	81.5	63.4	**66.9**	61.6	60.5	66.8	**70.5**
2	81.6	**82.7**	79.4	**88.5**	87.7	87.6	67.5	**69.8**	64.3	64.5	70.3	70.7
3	**92.3**	89.5	85.6	**92.6**	91.7	87.6	76.5	76.5	84.6	73.6	74.6	**76.4**

Significant values are in bold.

**Table 3:** Machine learning classification accuracy in multi-distance and multi-subject.

Subjects	4.5 m			3.5 m			2.5 m			2 m		
	RSSI (%)	Phase (%)	FeatureSet (%)	RSSI (%)	Phase (%)	FeatureSet (%)	RSSI (%)	Phase (%)	FeatureSet (%)	RSSI (%)	Phase (%)	FeatureSet (%)
SVM accuracy												
1	**80.2**	78.4	70.5	**85.4**	84.5	75.6	**76.5**	74.8	70.6	66.7	**67.6**	65.5
2	81.6	**82.7**	73.7	**90.5**	85.7	81.6	**76.5**	73.6	72.7	**66.6**	65.5	63.8
3	**89.4**	89.6	82.6	**91.6**	89.6	84.5	81.5	**83.6**	77.6	70.4	**72.9**	68.4
RF accuracy												
1	**73.7**	71.6	71.0	**76.6**	74.2	73.3	**69.3**	66.9	65.5	61.4	59.4	**63.4**
2	**81.2**	73.2	74.5	79.3	**81.4**	73.4	**71.8**	66.4	70.3	**65.5**	63.5	63.9
3	**81.7**	78.7	76.3	**90.8**	85.4	81.4	**78.6**	78.5	73.5	**67.7**	66.5	66.9
DT accuracy												
1	73.5	70.5	**77.4**	74.7	75.6	**78.4**	70.5	63.2	**68.6**	**66.3**	60.3	63.5
2	77.4	75.5	**81.4**	79.5	77.4	**80.5**	**73.4**	67.5	71.6	**65.5**	62.6	64.6
3	85.6	81.3	**85.4**	79.2	**82.3**	82.6	**79.8**	74.7	76.4	68.6	**70.8**	66.4

Significant values are in bold.

### Artificial intelligence model development

The development of an artificial intelligence (AI) model aims to provide assistance and support to individuals with limited mobility or disabilities, enabling them to perform daily activities in a contactless manner. One of the key challenges in developing an AI model for contactless AAL is ensuring the privacy and security of the user’s data.

#### Data analysis using DL models

To assess the efficacy of the system, experiments were conducted on datasets collected from a scenario-based environment. Only the front layers of the RSSI and phase profiles were used for all activities. The system’s performance was evaluated using three distinct DL models: long short-term memory (LSTM), convolutional neural network (CNN), and a combination of the two (LSTM+CNN). Before training an LSTM-CNN network, a raw RSSI data were transformed into a stack of matrices with dimensions of $5 \times 3$, corresponding to the tag array size. The experiment data was simplified by focusing on the 1D layer rather than converting to three levels using the timestamp dimension. The instantaneous RSSI value for each RFID tag was entered into the *i*-th row and *j*-th column of a matrix. If certain tags failed to provide RSSI readings due to blockage caused by human activity, the corresponding value defaulted to zero. The processed data was normalised with a mean of zero and a standard deviation of one before being passed into the network as input. The proposed hybrid DL models incorporated a single LSTM layer with a single dropout and flatten layer, while the CNN model utilised a 1*D* convolution layer due to the linear data structure. Two equally sized 1*D* convolutional layers and two identically sized *max pooling* layers were used, with a dense layer employed between the *flatten* and output layers. For the third model, fully connected layers were used to merge the LSTM and CNN models. The optimizer used was *adam*, with a decay of $1e^{-6}$ and a learning rate of 0.01. The activation function was set to *tanh*. The models were trained across 50 epochs, with user recognition being addressed as a problem of multi-class classification. The parameters tracked during the training included accuracy and loss.

#### Data analysis using ML models

In this study, we applied three classical ML algorithms, namely SVM, random forest, and decision tree classifier alongside DL algorithms to evaluate the collected dataset. We assessed accuracy using a train-test split technique, ensuring predictions were generated from data not used during model training. The data was divided into training and testing subsets with a train-test ratio of 0.8, meaning 80% of the data was used for training and the remaining 20% for testing, as detailed in Table 4.

**Table 4:** Hyper-parameters of ML/DL algorithms.

S. No.	Algorithms	Hyper parameters
1	LSTM	Optimizer = adam, hidden-layers-activation = tanh, lr = 0.01, loss = binary-crossentropy, batch-size = default, hidden-layer-size (15, 50), dropout = 0.2, out-layer-activation = softmax, epochs = 50
2	CNN	Optimizer = adam, hidden-layers-activation = tanh, lr = 0.01, loss = binary-crossentropy, batch-size = default, hidden-layer-size (15, 32), max-pooling-layer-size = (3, 1), out-layer-activation = softmax, epochs = 50
3	LSTM + CNN	Optimizer = adam, hidden-layers-activation = tanh, lr = 0.01, loss = binary-crossentropy, batch-size = default, LSTM-hidden-layer-size (15, 50), CNN-hidden-layer-size (15, 50), dropout = 0.2, max-pooling-layer-size = (3, 1), out-layer-activation = softmax, epochs = 50
4	SVM	Degree = 3, gamma = auto, kernel = linear, tol = 0.001, shrinking = true, C = 1.0
5	RF	n-estimator = 10, criterion = gini, max-features = auto, min-samples-leaf = 1, min-impurity-split = $1e-^{07}$, n-jobs = 1
6	ET	min-samples-leaf = 1, splitter = best, min-impurity-split = none, criterion = gini

### User recognition’s overall performance

**Figure 1:**
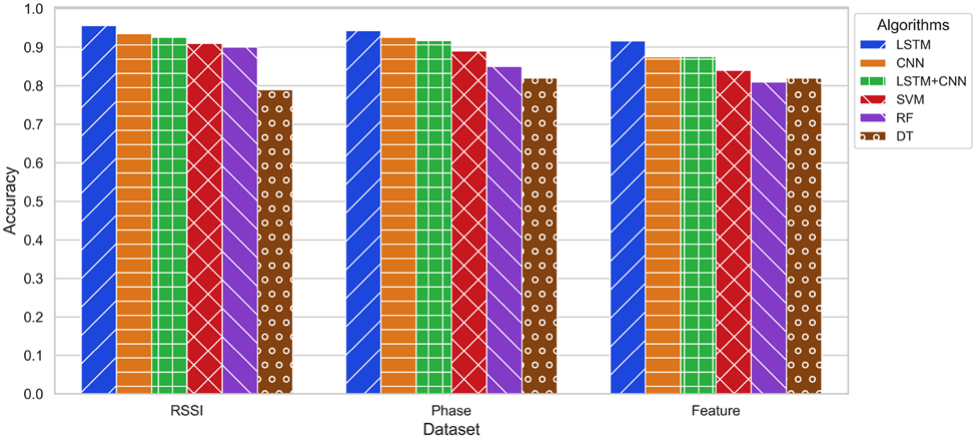
Comparison of DL and ML algorithms across various scenarios and approaches.

**Figure 2:**
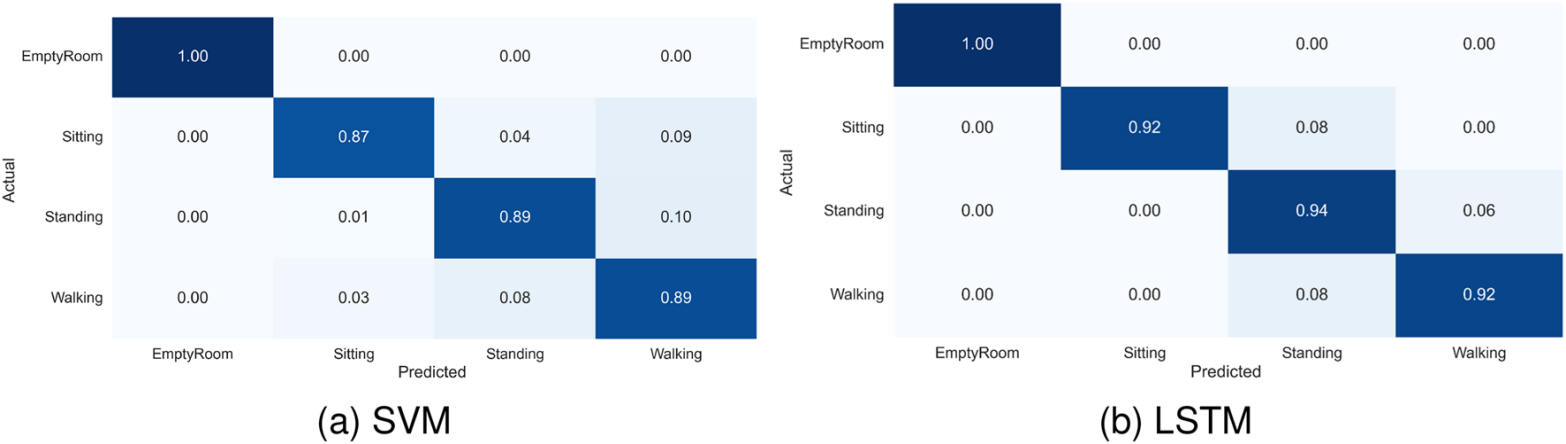
A normalized confusion matrix of various activities recognition using SVM and LSTM.

To evaluate the effectiveness of user recognition with our collected dataset, we employed the k-fold cross-validation technique. This method involves dividing the dataset into k equal-sized groups and randomly shuffling them. In each fold, k $-1$ groups are used for training, while the remaining group is used for validation. The process is repeated k times, and the average of the outcomes serves as the final estimate. We chose k = 5, resulting in each fold consisting of eight samples from each user for testing (240 samples total; $20\%$), while the remaining samples (960 samples total; $80\%$) are used for training. Thus, each sample is used exactly once as validation data across all folds. The overall results and normalised confusion matrix are depicted in Figs. 1 and 2.

## Discussion

This study assessed six algorithms, namely LSTM, CNN, LSTM+CNN, SVM, RF, and DT, for contactless RFID-based HAR across four distinct scenarios, employing three different approaches as detailed in “Evaluation and results”. The results revealed that in scenario 1, where the reader and antenna were 2 m from the subject and the subject-TRT-Wall distance was 0.5 m, DL algorithms (LSTM, CNN, and LSTM+CNN) outperformed the ML algorithms (SVM, RF, and DT). In contrast, in scenario 2, characterised by noise and a weak line of sight (LoS), the ML algorithms performed better, with SVM achieving the highest accuracy of $83.6\%$. In scenario 3, which entailed a strong NLoS environment, the DL algorithms, particularly LSTM, outperformed the ML algorithms, achieving an impressive accuracy of 95.6%. In scenario 4, where the reader and antenna were positioned 4.5 meters from the subject, the DL algorithms again exhibited better performance, with LSTM achieving the highest accuracy of $93.7\%$. Cross-validation further affirmed the reliability of both SVM and LSTM algorithms, achieving average accuracies of $91.6\%$ and $95.6\%$ respectively, as demonstrated in Fig. 2. The normalised confusion matrix underscored the LSTM model’s consistent recognition accuracy, surpassing $91\%$ for all subjects. This observation reinforces the potential of the proposed approach for practical implementation in user recognition applications, as depicted in Figs. 1, 9, and 11. Overall, the performance of DL and ML algorithms for contactless RFID-based HAR depends on the distance from *TRT-Wall* to the reader antenna. DL algorithms are better suited for scenarios requiring the capture of temporal dynamics of human activities, while ML are more effective in noisy environments or when the distance is limited.

## Data and methods

**Figure 3:**
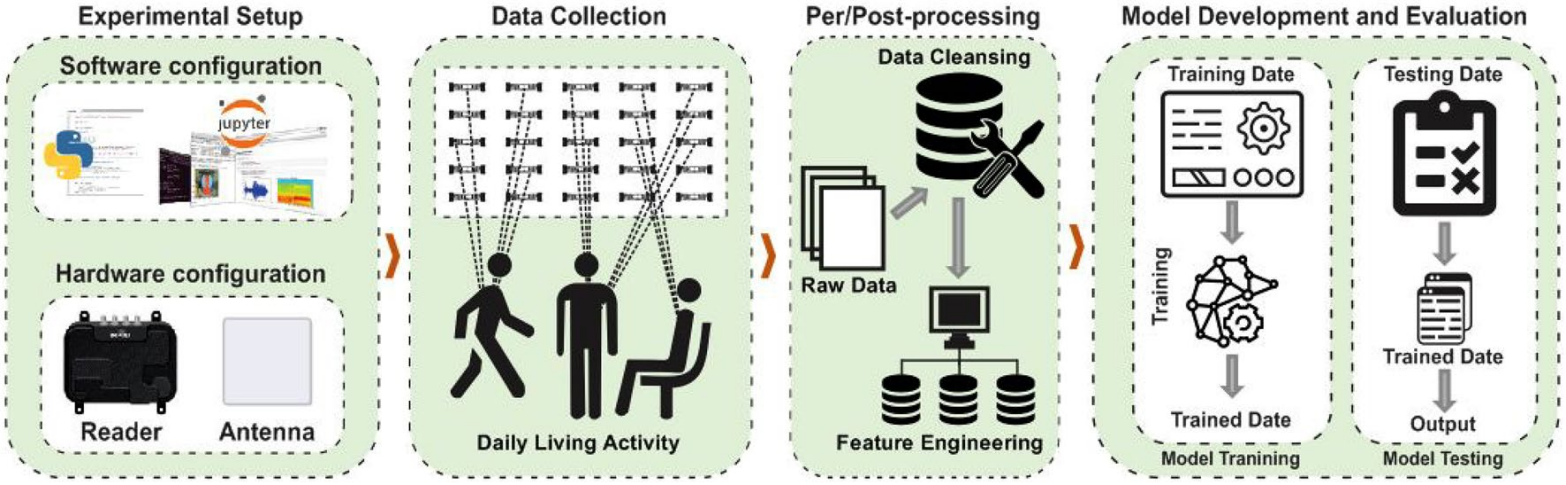
Data flow diagram: RSSI and Phase capture for human activity, dataset compilation for ML/DL classification.

This section presents the methodologies and materials used in an experimental setup involving multiple test scenarios before applying ML and DL techniques for predictive analytics. The hardware and software components were carefully organised to collect RSSI and Phase information from the RFID UHF passive tags array using reader sensing devices to indicate human activity. These components are detailed in subsections “Hardware setup” and “Software setup”. Our proposed methodology is depicted in Fig. 3, comprising four major components elaborated upon below.

### Experimental setup

The experiments presented in this paper, conducted within a $$10 \times 10$$ m$$^2 $$ room in the James Watts South building at the University of Glasgow, received ethical approval from the University of Glasgow’s Research Ethics Committee (approval nos.: 300200232, 300190109). All methods followed the guidelines and regulations of the Research Ethics Committee, and all subjects gave written informed consent before the data collection.

The experimental setup was designed to simulate a realistic indoor environment to assess the efficacy of our AAL system. The *TRT-Wall* setup mimics a typical room structure with dimensions of $1.5 \times 1.5$ m$^2$. The environment included multiple metal storage boxes and tables, providing rich multipath characteristics and a strong non-line-of-sight (NLoS) environment. The *TRT-Wall* is divided into five columns, each consisting of three tags, and three rows, totaling fifteen tags.

**Testbed configuration:**

1. Tags Arrangement: The tags are uniformly placed in a $3 \times 5$ grid, each 30 cm apart.
2. Antenna Placement: The circularly polarized antenna was placed at horizontal distances of 2, 2.5, 3.5, and 4.5 m from the center of the *TRT-Wall*. The height of the antenna was maintained at 0.75 m above the floor surface.
3. Subject Positioning: The subjects were positioned 0.5 m away from the *TRT-Wall* and instructed to perform various activities (sitting, standing, walking) at designated locations within the testbed.

To better demonstrate the experimental setup, we have included an additional figure that depicts the overall configuration and layout of the testbed.

#### Hardware setup

**Figure 4:**
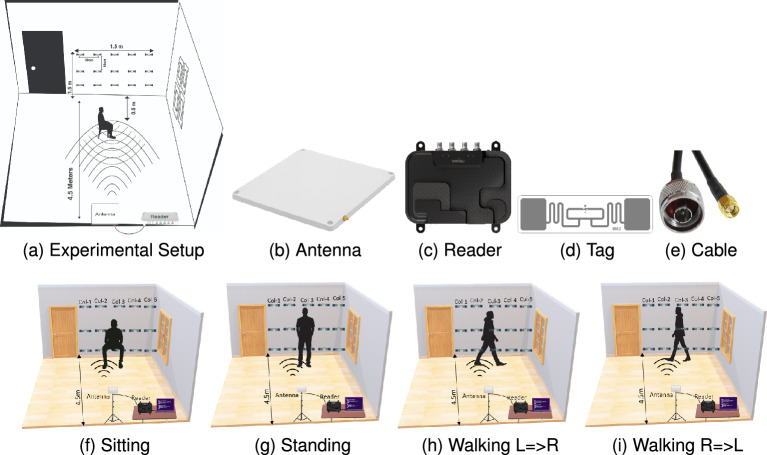
Experimental setup in a mocked room for activity recognition and localisation using TRT-Wall.

The proposed *TRT-Wall* for the AAL system utilises COTS UHF Gen-2 RFID devices without any hardware or firmware modifications. The system comprises a UHF passive RFID tag array and an *Impinj *$\textit{R700}$ reader (see Fig. 4c). The reader operates between 865 and 868 MHz using time-division multiplexing mode, can read up to 1100 tags per second, and is compliant with the EPC Class 1 and Gen 2 standard tags. These tags are attached to a board configured in a $3 \times 5$ grid, uniformly placed 30 cm apart, numbered from 1 to 15, and arranged from left to right, and top to bottom. A circularly polarised antenna with dimensions of 250 mm $\times$ 250 mm $\times$ 14 mm (see Fig. 4b) and an 8.0 dBi gain is connected to the reader. The wavelength $\lambda$ is set at 0.34 m, and the RF transmitter power is set to 30 dBm. The model training backend module runs on a laptop with an Intel^®^ Core $i7-10850H$ CPU at 2.7 GHz, dual-core, and 16 GB of RAM.

#### Software setup

To collect data, the reader’s data collection program is run on a laptop using the Impinj ItemTest Software version 2.8.0 (available at https://support.impinj.com). The process involves the reader interrogating tags repeatedly and capturing RSSI and phase information from the back-scattered signals. The transmitter then transmits the received measurements of RSSI and phase information from the tag array through a laptop’s RS232 serial port continuously. The streamed readings are received by the backend module and processed accordingly.

### Data collection and preprocessing

This section delineates the methodology employed for data collection. Firstly, we detail the various scenarios considered for data collection. This study has considered four distinct test scenarios, which are explained below.

1. **Test Scenario 1:** One subject performing activities with the reader and antenna positioned 2 m from the subject, and the subject placed 0.5 m away from the TRT-Wall.
2. **Test Scenario 2:** One subject performing activities with the reader and antenna positioned 2.5 m from the subject, and the subject placed 0.5 m away from the TRT-Wall.
3. **Test Scenario 3:** Three subjects performing activities with the reader and antenna positioned 3.5 m from the subject, and the subject placed 0.5 m away from the TRT-Wall.
4. **Test Scenario 4:** Three subjects performing activities with the reader and antenna positioned 4.5 m from the subject, and the subject placed 0.5 m away from the TRT-Wall.

#### Data collection

This study considered three subjects with varying ages, height, and weight to conduct experiments. To maintain consistency in both training and testing data, subjects performed four activities: sitting, standing, and walking (forward and backward) at their natural pace between the antenna and the *TRT-Wall*, as depicted in Fig. 4. Every subject provided informed consent, authorised by the University of Glasgow’s institutional review board. Data collection for each scenario involved subjects completing all activities while maintaining proximity to the *TRT-Wall* and antenna. Twenty samples, including RSSI and phase information, were collected per activity for each subject. Only one subject performed each activity at a time, as recognising multiple subjects simultaneous was not the study’s intention. Consequently, the data matrix contains information from 15 tags. Data was collected at distance of 2, 2.5, 3.5, and 4.5 m from the antenna. The inclusion of three subjects aimed to enhance diversity in the dataset. A total of 1200 valid training and testing samples were collected across four scenarios, with each tag read approximately 30–36 times during a 3-s interval. These raw RSSI and phase readings were parsed using a Python script to extract relevant information for further preprocessing before being used for training and testing various ML/DL algorithms. A summary of the collected dataset is presented in Table 5.

**Table 5:** Dataset summary using *TRT-Wall*: scenarios, subjects, and activities performed.

Activity	4.5 m		3.5 m		2.5 m		2 m	
	RSSI	Phase	RSSI	Phase	RSSI	Phase	RSSI	Phase
Empty room	20	20	20	20	20	20	20	20
Sitting	20	20	20	20	20	20	20	20
Standing	20	20	20	20	20	20	20	20
Walking forward	20	20	20	20	20	20	20	20
Walking backward	20	20	20	20	20	20	20	20

#### Data preprocessing

Data preprocessing is an essential step in analysing raw RSSI data, as it involves cleaning, formatting, and transforming the data into a structured format for further analysis. We used mathematical/statistical techniques such as moving average window and signal processing methods, including bandpass, low-pass, and high-pass filters, to focus on specific pattern. Initially, we processed the data using the following mathematical expression.

1 $$\begin{aligned} T_{f}\frac{y(k)-y(k-1)}{T}+y(k)=x(k), \end{aligned}$$

2 $$\begin{aligned} y(k)=\frac{T}{T_{f}+T}x(k)\frac{T_{f}}{T_{f}+T}y(k-1)=ax(k)+(1-a)y(k-1). \end{aligned}$$

The collected data for each activity was formatted into a 2D matrix, where each row contained an observation and the columns represented the corresponding RSSI and phase tags. To ensure data quality, we applied pre-processing functions using libraries (i.e., *Scikit* and *Pandas*). The data for each activity was stored in a matrix with 540 columns of RSSI data (15 tags x 36 columns) when the activity was performed without any blockages. To ensure robust and unbiased training of ML/DL models, synthetic data was generated using generative adversarial network (GAN) and conditional tabular generative adversarial network (CTGAN), standardizing the number of samples for each activity class^35^. The phase and RSSI data for activity were saved as separate data files with a *frame size* of 36 (representing approximately 36 reading in three seconds). During activities such as sitting or standing, the corresponding tags were either partially or fully read. To maintain a 36-time tag reading, any missing data for each tag was replaced with zeros and included in the data matrix. The analysis revealed that no tag was read more than 36 times, and missing *NaN* values were imputed with the mean of each row using the *SciKit* *SimpleImputer* function. Additionally, the pandas *unique* function was used to divide the timestamp into seconds and verify the correct reading of each tag for three seconds.

### Activity recognition using RSSI

The use of passive UHF RFID tags in indoor activity localisation for AAL is facilitated by a reader employing air interfaces protocol such as EPC class1 Gen-2 and ISO-18000-6c for data transmission and reception^36^. Within the realm of passive tag-based AAL, the RSSI RF capability can be effectively harnessed with COTS readers. In practical applications, passive RFID tags provide the reader with raw data in a 5-tuple format: RSSI, timestamp, EPC, TID, and frequency. The process of creating an RSSI dataset involves several steps outlined in Algorithm 1. The transformation required for solving the indoor propagation path loss model, used in various studies on RSSI distance transformation, can be derived through a simplified derivation.

3 $$\begin{aligned} P_{\text{ ower } }\left( d_{\text{ istance } }\right) _{d B m}=P\left( d_0\right) _{d B m}-10 n \log \left( \frac{d}{d_0}\right) +X_{d B m}, \end{aligned}$$

4 $$\begin{aligned} d=10^{\frac{P\left( d_0\right) _{d B m}-P(d)_{d B m}}{10 n}}, \quad \forall \quad d_0=1 \text{ and } X_{d B m}=0 \end{aligned}$$

where $P(d_{0})_{dBm}$ is received power along the propagation path of relative distance *d*, and $P(d_{0})_{dBm}$ along the propagation path of reference distance $d_{0} (1m)$.

**Algorithm 1:**
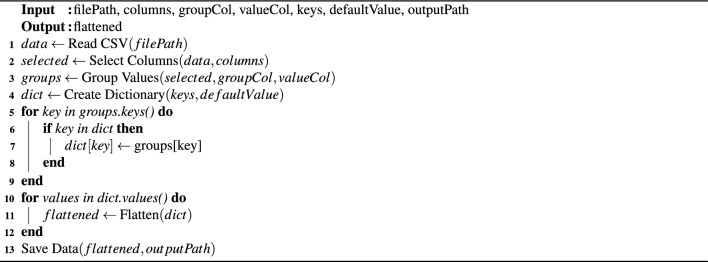
Pseudo code for RSSI dataset creation

**Figure 5:**
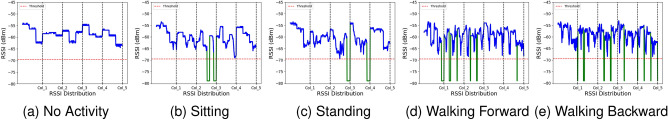
Illustration showcasing the diversity of activity recognition data through RSSI distribution and magnitude analysis.

The recognition of AAL was achieved through a series of carefully planned activities, as illustrated in Fig. 4. Specifically, five distinct activities were performed in the designated area in front of the *TRT-Wall*. For instance, the walking activity was conducted from the first to the fifth column, focusing on columns 3 and 4 for sitting and standing activity. Figure 5 shows the results, demonstrating that the RSSI variations can recognise each activity in the same location, with blocked tags causing RSSI drops. The RSSI strengths threshold was determined by observing maximum and minimum values, recorded at $-55$ dbm and $-69$ dbm respectively. The latter value, $-69$ dbm, was chosen as the threshold due to potential instances of non-reading or tag blocking. Instances of unread RSSI data or unrecognised activity are indicated by the colour green. Specifically, in Fig. 5a, the RSSI values for an empty room are displayed. Following this, Fig. 5b,c showcase sitting and standing activities in front of columns 3 and 4. Walking patterns from right to left and left to right are depicted in Fig. 5d,e, respectively.

#### RSSI-based walking direction analysis

**Figure 6:**
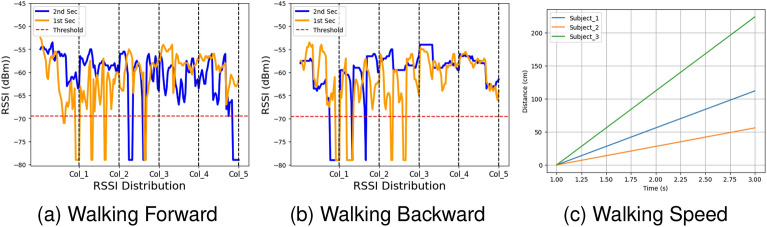
Illustration of walking speed and direction recognition via RSSI method with time split.

To clarify the walking direction, we partitioned the RSSI data into one-second intervals and distinguished each interval using two colours, as shown in Fig. 6. In Fig. 6a, during the 1-st second (in orange), the subject walked from column-1 to column-5, and during the 2-nd second (in blue), they moved from column-2 to column-5. In Fig. 6b, during the 1-st second, the subject walked from column-5 towards column-1, indicated by the colour change from column-5 to column-3, while during the 2-nd second, they walked from column-2 to column-1. This partitioning and color-coding approach effectively illustrates the walking direction in the RSSI data.

#### Walking speed estimation

To accurately estimate walking speed, we acquired RSSI measurements at 3-s intervals and collected data from three subjects. Preprocessing was performed to address noise issues and enhance the precision of speed calculations. This preprocessing step included the removal of outliers, filtering out unreliable readings, and applying smoothing techniques to mitigate the impact of measurement fluctuations caused by environmental factors. Walking speed is calculated by analyzing changes in the distance over these 3-s intervals, as depicted in Fig. 6c. This speed calculation is performed using a basic geometric formula: $\text {v} = \frac{\Delta \text {d}}{\Delta \text {t}}$.

### Activity recognition using phase

The utilisation of RF backscatter enables the signal to traverse a distance of 2*d* in dual directions, facilitating the monitoring of human activity through the analysis of RF phase differences using cross-relationships. The subsequent formula elucidates the correlation between distance, antenna phase rotation, and tag phase rotation:

5 $$\begin{aligned} \theta =\left( 2\pi \frac{2d}{\lambda }+\theta _{Ant}+\theta _{Tag}\right) \text {mod}(2\pi )\lambda . \end{aligned}$$

The phase is a periodic function of $2\pi$ radians occurring every $\lambda /2$ in the RF communication distance. The rotations of the antenna and tag phases are described by $\theta _{Ant}$ and $\theta _{Tag}$, respectively.

**Figure 7:**
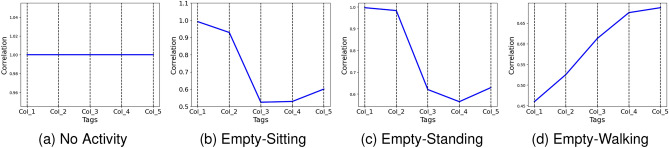
Illustration of activity recognition using Phase difference with co-relationship.

Assessing the precision and discriminatory nature of phase difference calculations during activity is essential. The significance is demonstrated in Fig. 7, which displays phase difference patterns during sitting and empty room in front of columns 3 and 4 using numpy’s *np.corrcoef(x,y)*. The cross-relationship function is explained in Algorithm 2. The co-relationship difference pattern suggests an effective method for modeling activities. The smooth variation of phase differences across blocking tags during sitting highlights their accuracy and reliability. The visualisation results indicated that the calculated phase differences are reliable and sensitive to AAL activity. To quantify the strength of the relationship between two different activities performed against the same tag (each tag has 36 phase reading values), the following formula can be used to calculate the correlation coefficient:

6 $$\begin{aligned} r_{xy} = \frac{{}\sum (x_i - \overline{x})(y_i - \overline{y})}{\sqrt{\sum (x_i - \overline{x})^2 \sum (y_i - \overline{y})^2}}, \end{aligned}$$

where $r_{xy}$ represent the correlation coefficient of the linear relationship between the tag value of empty activity and sitting activity tags, $x_i$, $y_i$ the values of the empty and sitting activity tags values whereas $\overline{x}$ and $\overline{y}$ denotes the mean of the values respectively.

**Algorithm 2:**
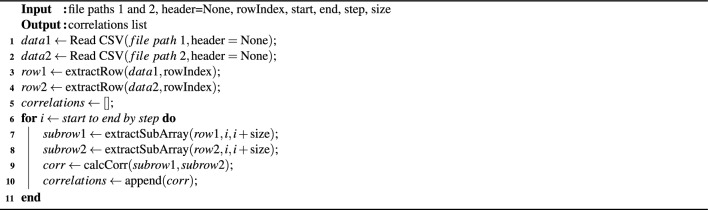
Pseudo code for phase correlation

### Activity recognition using FeatureSet

**Figure 8:**
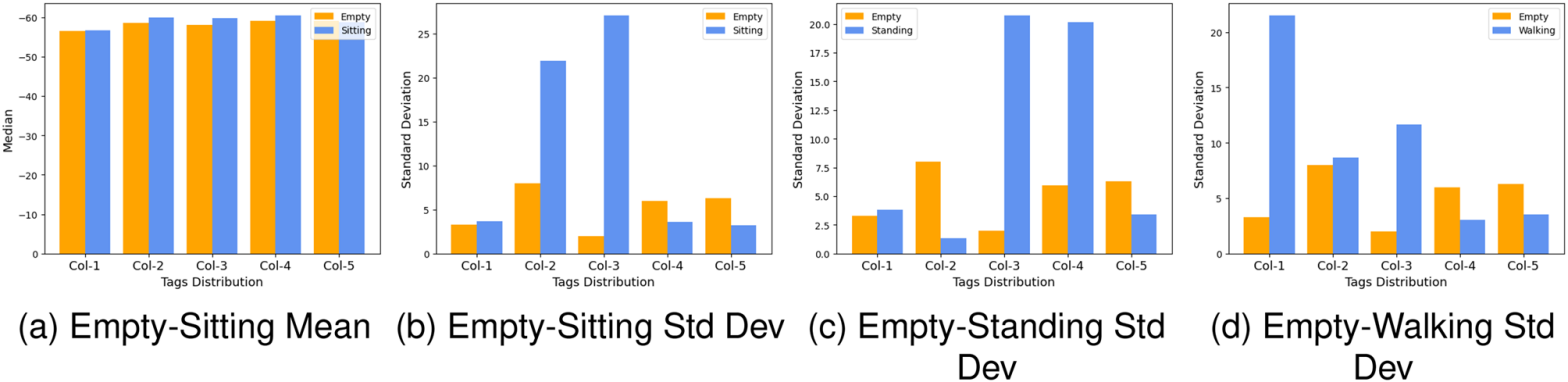
Illustration of activity recognition using standard deviation on the feature set.

The performance of AI models can be significantly enhanced through feature engineering. Flexible features allow for simpler, easier-to-maintain models, leading to better performance. Moreover, feature engineering reduces the time required for extracting numerous variables. This research focuses on processed matrix data, with rows indicating activities or occurrences. Each activity has 540 columns, with each row including 36 RSSI/phase samples (frame sizes) collected using 15 tags over a 3-s timeframe. The objective is to extract high-order features from the CSV file to significantly reduce data dimensions and boost the system’s robustness and classification accuracy. By combining statistical features such as mean, median, mean absolute value, standard deviation, variance, minimum, maximum, skewness, kurtosis, count, entropy, trimmed mean, trimmed variance, trimmed minimum, trimmed maximum, trimmed standard deviation, trimmed standard error, variation, score at percentile, and correlation coefficient, the study determines the optimal feature subset for AAL classification. Figure 8 demonstrated activity recognition by highlighting the standard deviation between empty activity and sitting, empty and standing, and empty and walking.

### Ablation studies

The section systematically investigated key factors influencing RFID-based human activity recognition. It concluded that maintaining a 0.5 m proximity between *TRT-Wall* and antenna is optimal. Additionally, it highlighted the importance of reducing the number of tags for more efficient data transmission. The study also explored the influence of subject quantity on detection accuracy, revealing a non-linear relationship. Lastly, it emphasized the critical role of antenna height in optimising system performance. It concluded that a default height of 0.75 m ensures robust outcomes.

#### Impact of distance from *TRT-Wall* to antenna settings

**Figure 9:**
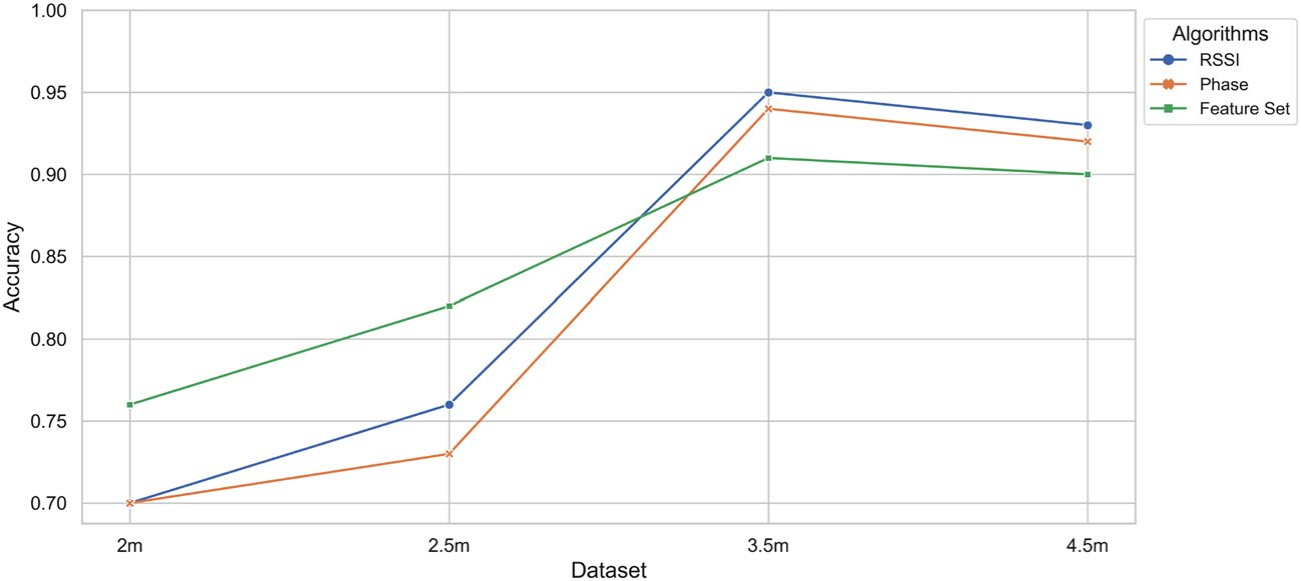
Assessing LSTM accuracy through four different scenarios and approaches.

The study adopts the *TRT-Wall* approach, employing an array of tags to decouple the activity recognition of subjects. Throughout the experiments, accuracy is measured at different distances between the antennas and tags, spanning from 2 to 4 m. The results reveal a statistically significant correlation between the *TRT-Wall* distance and the antenna. Notably, a *TRT-Wall* distance of 1 and 2 m causes severe distortion in RSSI and Phase waves, resulting in false positives during recognition. The initial tag distance between the subject and the *TRT-Wall* is set at 0.5 m. Subsequently, the accuracy of activity recognition is further tested at various subject-to-antenna distances, ranging from 2 to 4.5 m. The results indicate that the accuracy decreases with decreasing distance for unobstructed readings and beyond 3.5 m, factors such as weak signals and the lower reading rate of RFID tags affect accuracy. To address the impracticality at shorter distances, the proposed study maintains a default setting of 0.5 m between tags and subjects while testing the distance between the subject and the antenna at 2, 2.5, 3.5,  and 4.5 m (Fig. 9).

#### Impact of number of tags

**Figure 10:**
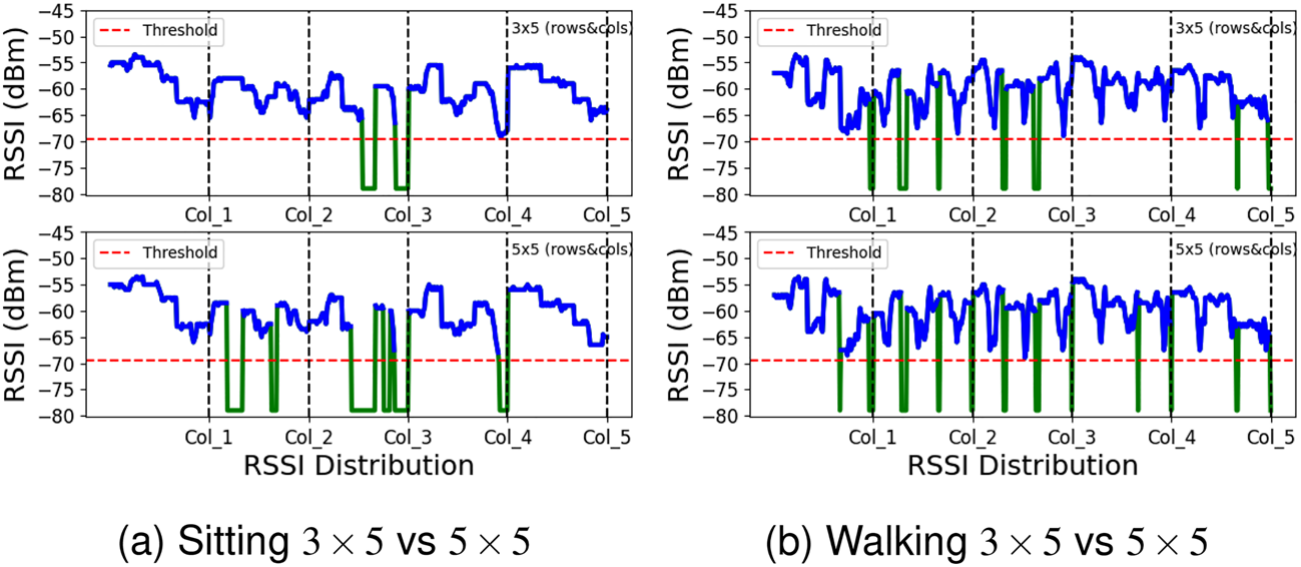
Impact of number of tags.

Increasing the number of tags leads to a greater number of reflected signals, broader pathway coverage, and enhanced data collection for activity detection. Our experiments reveal that a reduced tag count facilitates efficient coupling and transmission of activity data. By eliminating two rows containing five tags each, we reduce the total tag count down from 25 (arranged in 5 rows and 5 columns) to 15 (arranged in 3 rows and 5 columns). This reduction not only provides supplementary information but also enhances activity prediction performance, as depicted in Fig. 10a illustrating sitting activity and Fig. 10b showcasing walking activity. If the goal is to augment the environmental path complexity, it is advisable to increase the number of tags in the columns. Additionally, within indoor environments, the incremental cost of adding more tags is marginal compared to the expense of incorporating additional readers with antennas^37^. To implement this, our study employs a default configuration comprising a single circularly polarised antenna and 15 passive UHF tags.

#### Impact of number of subjects

**Figure 11:**
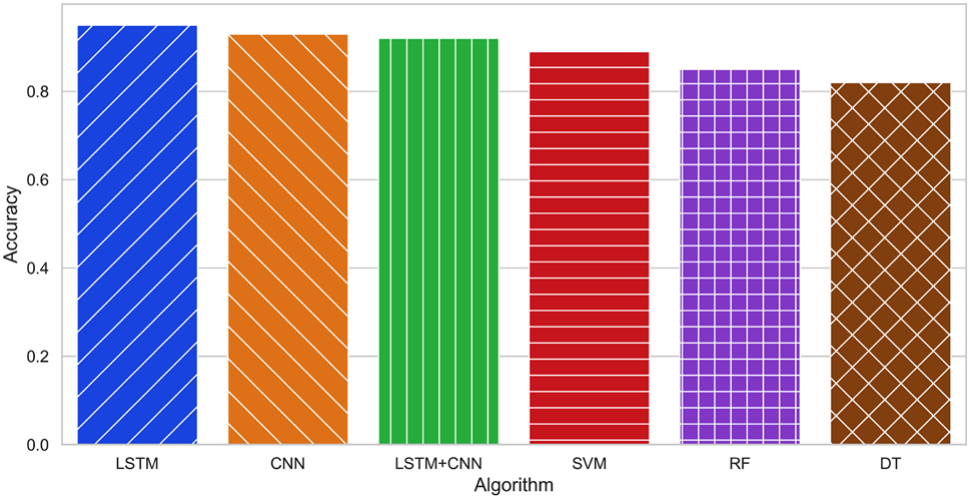
Scenario 3: a comparison of DL and ML algorithms on 3.5 m.

This section examines how the number of subjects affects performance and concludes that detection accuracy does not increase linearly with the number of subjects, but rather gradually. Since fewer subjects indicate less variation in user characteristics, this result is consistent with our expectations. We assess a number of parameters in our study, including RSSI, Phase, and feature values. When there are three subjects involved, our suggested method has an accuracy of $96\%$, but when there is just one subject, it only has an accuracy of $81.20\%$. Nevertheless, WiFi-ID^38^ and WiWho^39^ outperform our method when detecting six subjects at a single distance using Wi-Fi signals, achieving accuracy levels of $77\%$ and $80\%$, respectively. As shown in Table 6, RFree-ID^40^, TagFall^24^, and DFD^41,42^ achieved high accuracy levels of $93\%$, $94\%, 94\%$, and $95\%$ for detecting human activities such as walking or falling using RFID. It needs to be noted that it may not be practical or convenient to enable subjects to perform activities at a distance of 4.5 m for proper recognition (Fig. 11).

**Table 6:** Analysis of different user activity detection approaches.

Approach	Technology	Group size	Antenna	Accuracy (%)
WiFi-ID^38^	WiFi	6	1	77
WiWho^39^	WiFi	6	3	80
WiPg^43^	WiFi	5	3	92.7
RF-Motion^29^	RFID	1	4	90
RFree-ID^40^	RFID	5	1	93
TagFall^24^	RFID	1	4	94
DFD^41^	RFID	1	1	94
RF-Car^34^	RFID	1	4	95
**TRT-Wall**	*RFID*	*3*	*1*	**95.6**

Significant values are in bold.

Significant values are in italics.

#### Impact of antenna height

This study examines how the performance of RFID tag reading systems is affected by antenna height. The system’s range and precision are significantly influenced by the antenna height. Generally, increasing the antenna height can improve system performance. Nonetheless, we conducted an experiment to examine three different antenna placement scenarios in our RFID tag reading system. In the first case, the antenna was mounted against a $1.5 \times 1.5$ m$^2$ wall at ground level. Our research showed that the tags on the top row were not properly read, which reduced the recognition accuracy. In the Second scenario, we adjusted the antenna height to 0.75 m, placing it at the centre of the wall and ensuring a LoS. It was thought that a small change in antenna height would have little impact on accuracy. Nevertheless, we noticed a decrease in the tag signal strength when the antenna height was raised further from 0.75 to 1.5 m. The was due to the reader’s signal was not strong enough to reach the lower row of tags. According to the findings of our experiments, maintaining the default antenna height of 0.75 m, or mid-height of the wall, is necessary to achieve optimal system performance. After experimental verification, it is clear that the system is robust for AAL.

## Limitations and future directions

The *TRT-Wall* approach is a key step toward enabling the accurate detection of indoor activities without requiring users to wear or carry any RFID tags. Nonetheless, there are numerous opportunities to further strengthen the basic TagFree concept in the future.

### System flexibility

The study investigated the precision with which *TRT-Wall* can detect each subject’s activity. Nonetheless, it is expected that the system’s scope could expand to monitor multiple subjects simultaneously due to complex subject interactions. This would require more extensive data pre-processing and analysis. According to our preliminary study, deep learning algorithms are better at detecting individual activities than conventional methods. Moreover, the *TRT-Wall* range is limited to 12 m when using a single antenna. This reading range can be extended by installing larger antenna arrays using an Impinj antenna hub and employing many RFID tags as references for wider coverage.

### Model generalisation

The implementation of our proficient deep learning model is limited to non-uniform antenna configurations and tag placements. Consequently, the model necessitates retraining to accommodate heterogeneous environments. An alternative approach to enhance predictive outcomes involves tuning the model through federated learning. Federated learning facilitates training on various various samples, presuming that certain activities occur in a spcecific sequential order. This results in a generalised and uniform trained model that is adaptable to any heterogeneous environment.

### User authentication

The ability of the current approach to distinguish between a limited number of activities and user detection is restricted. In the future, this limitation could be overcome by developing a user identification and recognition model that utilises strong and complete user attributes, rather than focusing solely on activities.

## Conclusions

This study utilises multipath signals generated by various activity patterns collected from commercial Impinj RFID readers to establish a cost-effective, contactless, and privacy-preserving user identification mechanism. We demonstrate the efficacy of our system, *TRT-Wall*, in identifying activities in typical indoor environments without tagging the targets. We employ a data preprocessing technique in our tag-free model, which gives comprehensive information for activity recognition. The tag-free activity detection problem is successfully addressed using a Long Short Term Memory network. Our comprehensive experimentation outcomes and the implementation with commercial RFID devices validated that our tag-free *TRT-Wall* approach outperforms existing state-of-the-art methods, achieving average activity identification accuracy rates of 95.6%, 94.3%, and 91.6% for RSSI data, phase difference, and features, respectively, with up to three subjects in multipath-rich environments.

## Data Availability

The datasets utilized in the current study are available from the corresponding author upon reasonable request at *m.khan.6@research.gla.ac.uk*.
